# A New Polyunsaturated Brominated Fatty Acid from a *Haliclona* Sponge

**DOI:** 10.3390/md7040523

**Published:** 2009-11-02

**Authors:** Satoe Aratake, Agus Trianto, Novriyandi Hanif, Nicole J. de Voogd, Junichi Tanaka

**Affiliations:** 1 Department of Chemistry, Biology and Marine Science, University of the Ryukyus, Nishihara, Okinawa 903-0213, Japan; E-Mail: k088302@eve.u-ryukyu.ac.jp (S.A.); 2 Department of Marine Science, Diponegoro University, Semarang 50234, Indonesia; E-Mail: trianto_telawur@yahoo.co.id (A.T.); 3 Department of Chemistry, Graduate School of Science, Nagoya University, Furo-cho, Chikusa-ku, Nagoya 464-8602, Japan; E-Mail: novriyandi@hotmail.com (N.H.); 4 Naturalis, National Museum of Natural History, PO Box 9517, 2300 RA Leiden, The Netherlands; E-Mail: voogd@naturalis.nnm.nl (N.J.d.V.)

**Keywords:** sponge, fatty acid, cytotoxicity

## Abstract

A new polyunsaturated brominated fatty acid possessing acetylenic bonds **1** was isolated from the Indonesian sponge *Haliclona* sp. The structure of compound **1** was elucidated by analyzing its spectral data. It showed moderate cytotoxicity against cultured cells.

## 1. Introduction

To date a number of linear acetylenic compounds have been isolated from marine organisms. Among them, sponges are the most prolific sources of molecules of this class. Representative members retain unique structures and show biomedical potential, *e.g.*, lembehynes showing neuritogenic activity [1], petrosynol and petrosynolic acid inhibiting reverse transcriptase of human immunodeficiency virus [2], pellynols showing potent cytotoxicity [3], and a thiophene-containing fatty acid showing antimicrobial activity [4]. In our quest to discover new bioactive molecules from marine invertebrates of the Okinawan and Indonesian coral reefs, we screened extracts prepared from specimens collected around Alor Island, Indonesia. A lipophilic extract of the sponge *Haliclona* sp. showed cytotoxicity against cultured cells, and hence, we isolated its active constituent, the structure of which is the subject of this manuscript.

## 2. Results and Discussion

The sponge was extracted with acetone. Its lipophilic extract was separated on a silica gel column, followed by reverse-phase HPLC to yield compound **1** as an oil, which gradually decomposes during handling. The molecular formula of the compound was C_20_H_21_BrO_2_ by ESITOFMS, indicating ten degrees of unsaturation. This could be accounted for by the presence of five olefins (ten signals from δ 110.1 to 140.9), two acetylenes (δ 68.9 d, 80.0 s, 83.6 s, 89.6 s), and one carboxylic acid (δ 174.0; 1,713 cm^−1^). Strong UV absorption at 256 nm suggested the presence of conjugated systems.

Four partial structures **i–iv** were revealed by inspecting COSY cross peaks (Table 1): (**i**) a diene (a vinyl proton at δ 6.67 (H-5) next to *cis* olefinic protons at δ 5.67 and 6.23) with a methylene at δ 3.20 (H-2), (**ii**) a double bond (δ 5.47 and 5.63; H-8,9) flanked by two methylenes at δ 3.39 (H-7) and 3.15 (H-10), (**iii**) conjugated *trans* double bonds (δ 5.52, 6.50, 6.13, and 5.79; H-13 to H-16) with a methylene at δ 2.31 (H-17), and (**iv**) a terminal acetylenic proton at δ 1.90 (H-20) with a methylene at δ 2.28 (H-18). The remaining substituted acetylene should be placed between units **ii** and **iii** because HMBC correlations H-10/C-11,12 and H-14/C-12 were observed. Additional HMBC correlations enabled us to connect the following units: **i** and **ii** (H-5/C-6,7 and H-7/C-5,6), **iii** and **iv** (H-17/C-18 and H-18/C-17), and **i** and the carboxylic acid (H-2/C-1). Bromine was placed at the sole quaternary olefinic carbon at C-6. NOE between H-7 and H-4 determined 5*E* configuration, while 8*Z* configuration was assigned by the *J* value (10.5 Hz) between H-8 and H-9 with decoupling experiments. Therefore, the entire structure was elucidated to be 6-bromo-icosa-3*Z*,5*E*,8*Z*,13*E*,15*E*-pentaene-11,19-diynoic acid. Although more than twenty fatty acids with bromination at C-6 have been reported [5–11], compound **1** had the highest unsaturation degree of this class.

We evaluated the cytotoxicity of the purified molecule **1** against NBT-T2 rat bladder epithelial cells, and the IC_50_ value was estimated to be 36 μg/mL. This weaker activity than the original extract was because of the presence of other toxic components and/or decomposition of the molecule during the assay.

## 3. Experimental Section

### 3.1. General Procedures

ESIMS was measured on a PE QSTAR mass spectrometer. FTIR and UV spectra were obtained on Varian FTS-3000 and Hitachi U-2000 instruments. ^1^H- and ^13^C- as well as 2D NMR spectra were obtained on a Jeol A500 spectrometer in CDCl_3_ with reference to an internal standard of TMS. Chemical shifts and coupling constants were expressed in δ and Hz.

### 3.2. Animal Material

**Figure 2.**
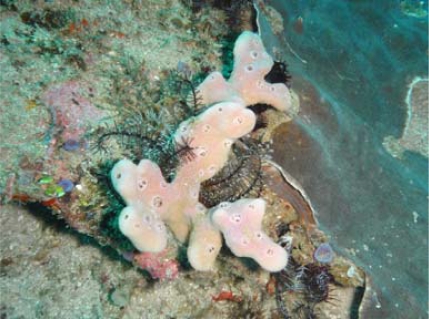


The sponge shown below in Figure 2 was collected at a depth range of 20–35 m by hand during scuba diving on the strait between Alor and Pantar Islands, Nusa Tengara Timur, Indonesia. The specimen was identified by one of the authors (NJdV) as *Haliclona* sp., Chalinidae, Haplosclerida, and deposited at Naturalis, National Museum of Natural History, Leiden, the Netherlands, with a code RMNH POR 4825.

### 3.3. Isolation of compound **1**

The frozen sponge (wet weight, 43.4 g) was cut and steeped in acetone (200 mL) three times. The combined acetone solution was concentrated under vacuum, and the resulting residue was partitioned between EtOAc and water. The organic layer yielded 365 mg of a crude oil showing cytotoxicity at 1 μg/mL. The extract was separated on a silica gel with stepwise elution (Hexane-EtOAc: 1–0, 10–1, 1–1, 0–1, and MeOH) to give six fractions. Of these, the fifth fraction (18.5 mg) eluted with EtOAc was further separated by reverse-phase HPLC (RP-18, MeOH-H_2_O, 50-1) to yield compound **1** (7.9 mg). An additional amount was obtained from recollected specimens.

### 3.4. Compound **1**

Oil. HRESIMS *m/z* 395.0604, 397.0601 (calcd. for C_20_H_21_BrO_2_Na, 395.0623, 397.0602); FTIR (neat) 3,295, 3,026, 2,925, 2,361, 2,214, 2,116, 1,713, 1,593 cm^−1^; UV λmax 256 nm (logɛ 4.5, MeOH); and ^1^H- and ^13^C-NMR see Table 1.

### 3.5. Cytotoxicity testing

NBT-T2 rat bladder epithelial cells (BRC-1370, purchased from Riken BioResource Center) were cultured in DMEM supplemented with 10% heat-inactivated fetal bovine serum and antimicrobial agents using a standard protocol and seeded in 200 μL wells. After preincubation (37 °C, 24 h), cells were exposed to graded concentrations of compounds in duplicate (37 °C, 48 h). The cells were treated with MTT solution (15 μL, 5 mg/mL in PBS) after removal of the medium and incubated for 3 h. Residual formozan was dissolved in DMSO (100 μL) and absorbance was measured using a Tecan Sunrise microplate reader at 560 nm. IC_50_ values were estimated by plotting absorbance values against concentrations.

## Figures and Tables

**Figure 1.**
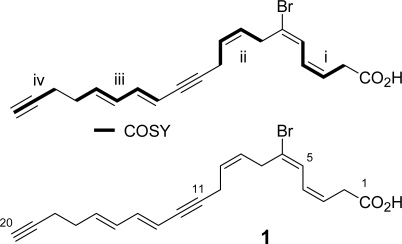
Structure of Compound **1**.

**Table 1.** ^1^H- and ^13^C-NMR Data (in CDCl_3_) for compound **1**.

**C#**	**δC**	**δH (mult.,*J*in Hz)**	**COSY**	**HMBC**
1	174.0 s			
2	32.8 t	3.20 (d, *J* = 6.8)	H-3	C-1, 3, 4, 7
3	125.3 d	5.67 (dt, J = 11.2, 6.8)	H-2, 4	C-5
4	125.4 d	6.23 (dd, *J* = 11.5, 11.2)	H-3, 5	C-2
5	127.1 d	6.67 (d, *J* = 11.5)	H-4	C-3, 6, 7
6	128.6 s			
7	34.2 t	3.39 (brd, *J* = 7.1)	H-8	C-2, 5, 6, 8
8	126.6 d	5.47 (dtt, *J* = 10.5, 1.7, 7.1)	H-7, 9	C-10
9	126.9 d	5.63 (dtt, *J* = 10.5, 7.1, 1.7)	H-8, 10	C-7
10	18.4 t	3.15 (brd, *J* = 7.1)	H-9, 13	C-8, 11, 12
11	89.6 s			
12	80.0 s			
13	110.1 d	5.52 (brd, *J* = 15.6)	H-10,14	C-15
14	140.9 d	6.50 (dd, *J* = 15.6, 10.7)	H-13, 15	C-12
15	130.9 d	6.13 (dd, *J* = 15.4, 10.7)	H-14, 16	C-13, 17
16	134.3 d	5.79 (dt, *J* = 15.4, 6.1)	H-15, 17	C-14, 17
17	31.6 t	2.31 (m)	H-16	C-15, 16, 18,
18	18.3 t	2.28 (m)	H-20	20
19	83.6 s			C-17, 19, 20
20	68.9 d	1.90 (t, *J* = 2.4)	H-18	
